# BUILDING LONG-TERM HIV SURVIVOR RESEARCH CHAMPIONS FOR PERSON-CENTERED CARE VIA COMMUNITY DISSEMINATION

**DOI:** 10.1093/geroni/igad104.3779

**Published:** 2023-12-21

**Authors:** Margaret Danilovich, Andy Rapoport, Meredith Nicholson

**Affiliations:** Northwestern University, Chicago, Illinois, United States; CJE SeniorLife, Chicago, Illinois, United States; GMHC, New York City, New York, United States

## Abstract

As HIV becomes a chronic condition, it is critical for research to include the perspectives of long-term HIV survivors who face the challenges of managing both HIV and aging. We created SHARE (Survivors of HIV Advocating for Research Engagement), a nationwide research advisory board for long-term survivors. SHARE’s goal is to incorporate long-term HIV survivor voices into research to influence person-centered care for long-term survivors. Through SHARE, board members have built research capacity through research methods training culminating in the board conducting a community needs assessment to learn community-directed priorities for HIV and aging. Following the needs assessment, each board member worked to identify and conduct research dissemination activities within their own communities to engage others in conversations about aging and HIV and educate on opportunities for greater research involvement. In this paper presentation, the speakers describe the process of building capacity among SHARE members so that they could serve as research champions through community dissemination efforts. The presenters will describe the methods of community dissemination including lunch and learns, webinars, infographics, podcasts, photovoice, and advocacy. Mixed-methods analysis of the impact of research dissemination will be presented including changes in community member knowledge about research, willingness to participate in research studies, and understanding of tools to find and use research. The presenters will also share data from SHARE board member self-reflection on the challenges and opportunities for researchers to engage community member in dissemination activities that are tailored to the community and can influence adoption of research among end users.

